# Geometric deep learning reveals a structuro-temporal understanding of healthy and pathologic brain aging

**DOI:** 10.3389/fnagi.2022.895535

**Published:** 2022-08-23

**Authors:** Pierre Besson, Emily Rogalski, Nathan P. Gill, Hui Zhang, Adam Martersteck, S. Kathleen Bandt

**Affiliations:** ^1^Department of Radiology, Feinberg School of Medicine, Northwestern University, Chicago, IL, United States; ^2^Advanced Neuroimaging and Surgical Epilepsy (ANISE) Lab, Northwestern University, Chicago, IL, United States; ^3^Mesulam Center for Cognitive Neurology and Alzheimer’s Disease, Feinberg School of Medicine, Northwestern University, Chicago, IL, United States; ^4^Department of Psychiatry and Behavioral Science, Feinberg School of Medicine, Northwestern University, Chicago, IL, United States; ^5^Department of Preventive Medicine, Feinberg School of Medicine, Northwestern University, Chicago, IL, United States; ^6^Helen Wills Neuroscience Institute, University of California, Berkeley, Berkeley, CA, United States; ^7^Department of Neurological Surgery, Feinberg School of Medicine, Northwestern University, Chicago, IL, United States

**Keywords:** geometric deep learning, brain shape, brain mapping, brain age, human aging, dementia, Alzheimer’s disease

## Abstract

**Background:**

Brain age has historically been investigated primarily at the whole brain level. The ability to deconstruct the brain into its composite parts and explore brain age at the sub-structure level offers unique advantages. These include the exploration of dynamic and interconnected relationships between different brain structures in healthy and pathologic aging. To achieve this, individual brain structures can be rendered as surface representations on which morphologic analysis is carried out. Combining the advantages of deep learning with the strengths of surface analysis, we investigate the aging process at the individual structure level with the hypothesis being that pathologic aging does not uniformly affect the aging process of individual structures.

**Methods:**

MRI data, age at scan time and diagnosis of dementia were collected from seven publicly available data repositories. The data from 17,440 unique subjects were collected, representing a total of 26,276 T1-weighted MRI accounting for longitudinal acquisitions. Surfaces were extracted for the cortex and seven subcortical structures. Deep learning networks were trained to estimate a subject’s age either using several structures together or a single structure. We conducted a cross-sectional analysis to assess the difference between the predicted and actual ages for all structures between healthy subjects, individuals with mild cognitive impairment (MCI) or Alzheimer’s disease dementia (ADD). We then performed a longitudinal analysis to assess the difference in the aging pace for each structure between stable healthy controls and healthy controls converting to either MCI or ADD.

**Findings:**

Using an independent cohort of healthy subjects, age was well estimated for all structures. Cross-sectional analysis identified significantly larger predicted age for all structures in patients with either MCI and ADD compared to healthy subjects. Longitudinal analysis revealed varying degrees of involvement of individual subcortical structures for both age difference across groups and aging pace across time. These findings were most notable in the whole brain, cortex, hippocampus and amygdala.

**Conclusion:**

Although similar patterns of abnormal aging were found related to MCI and ADD, the involvement of individual subcortical structures varied greatly and was consistently more pronounced in ADD patients compared to MCI patients.

## Introduction

The concept of brain age has been evolving since its introduction in the late 1960s (Oeriu, 1969). Advances in computational techniques and resources over the past 10 years have facilitated a dynamic expansion not only of our understanding of what brain age represents but how it can contribute to our understanding of healthy and disordered human aging. In the last 40 years, numerous studies have examined the relationship between neuroimaging-defined brain features and age using mass-univariate tests across brain voxels or regions. Hippocampal atrophy in both normal and pathologic aging patterns has been described across numerous investigations (Seab et al., 1988; Fox et al., 1996; Laakso et al., 1996; Jack et al., 1998). The temporal evolution of this atrophy may track age-related memory loss and aid in the diagnosis of various dementias, including Alzheimer’s disease dementia. Additionally, regional volume loss of the thalamus and putamen have been described in individuals with impaired memory (de Jong et al., 2008) while volume loss of peri-ventricular structures including the caudate, amygdala and thalamus have also been described (Ferrarini et al., 2006). Within the cortex, the regions suffering from volume loss are varied and include much of the neocortical regions involved in learning, memory and attention with cortical regions included in the default mode network the most consistently described as suffering from progressive atrophy in aging individuals (Kalpouzos et al., 2012).

Contrasted against the studies above that use age as an independent variable, brain age algorithms are trained to predict chronologic age based on features from thousands of individuals’ brain recordings or images. Many machine learning methods have been employed to predict age including linear and kernel regressions and deep learning. Examples for each include elastic net regression, relevance vector regression, or convolutional neural networks (CNN). Most prior brain age algorithms have used features derived from structural T_1_-weighted MR images (Franke et al., 2010; Gaser et al., 2013; Valizadeh et al., 2017; Wang J. et al., 2019). Less commonly, studies have predicted brain age with diffusion-weighted MRI (Mwangi et al., 2013; Tonnesen et al., 2020; Beck et al., 2021), functional MRI (Liem et al., 2017), MR angiography (Nam et al., 2020), FDG PET (Goyal et al., 2019), EEG (Sun et al., 2019; Paixao et al., 2020), or MEG (Engemann et al., 2020; Xifra-Porxas et al., 2021).

Despite the vast array of approaches used to determine brain age, most prior investigations have yielded a single per-participant prediction of brain age at the whole brain level, using an aggregate of all brain features at once. Popular approaches have used all structural MRI gray matter segmented voxels (Franke et al., 2010; Beheshti et al., 2018), while other studies have used regional thickness, surface area, and volume measurements (Kaufmann et al., 2019). More recent work have proposed morphological analysis using isosurfaces of functional brain imaging and successfully characterized neuropathologic-related features (Castillo-Barnes et al., 2020).

The ability to offer brain structure-specific analysis has been limited by the methods employed in previous investigations, specifically the analysis of the brain as a single structure yielding whole brain level predictions. This whole brain approach to aging has limited our ability to not only investigate the unique contribution of each individual brain structure or region to the aging process but also the role time plays in each structure’s contribution to aging.

Considering the brain not as a single structure but as a composite unit of multiple, dynamically interacting structures can offer a higher degree of granularity of exploration and understanding. The ability to deconstruct the brain into its component parts requires a different approach to analysis. Doing so requires the ability to extract information about each structure in isolation. Rendering individual brain structures as surface representations of their outer contour offers that opportunity. The investigation of brain morphology is a long-standing field of neuroscience. Van Essen posited that the brain’s shape, its fundamental sulcal and gyral patterns, are directly related to brain function due to underlying neuronal connectivity mounting similarly functioning brain regions into gyri and separating disparately functioning brain regions by sulci (Van Essen, 1997). Brain surface morphology has since been implicated in a variety of traits including personality, cognition and functional status (Whittle et al., 2009; Jockwitz et al., 2017; Cachia et al., 2018). Combining the advantages of CNN architectures with the strengths of brain surface analysis to advance Defferard’s graph CNN (gCNN) method into the neuroimaging domain (Defferrard et al., 2016; Besson et al., 2021), we developed surface based deep learning (SBDL) to fill this gap. Only a single prior investigation has explored structure-specific investigation of brain age and does so at a largely proof-of-concept level (Wachinger et al., 2015). In this pioneering work, which relied on the extraction of the eigenvalues of the Laplace-Beltrami operator calculated on the cortical surfaces and a selection of subcortical surfaces, the authors demonstrated that age could be accurately predicted using shape descriptors of individual brain structures.

Here, expanding our novel gCNN approach, we investigated individual structure age using a cross-sectional study design to assess structure age difference between healthy controls, MCI and ADD patients, and using a longitudinal study design to monitor the aging process of each structure and assess their dynamic implication in pathologic aging.

## Materials and methods

### Subjects

MRI data, age at scan time and diagnosis of dementia were collected from seven publicly available data repositories. Data used in the preparation of this article were obtained from seven publicly available repositories of neuroimaging data, including the Alzheimer’s Disease Neuroimaging Initiative (ADNI) database^1^, the details of which can be found in Table 1.

**TABLE 1:** Repartition of included subjects across datasets.

Dataset	Number of unique subjects	Number of scans	Age at scan (mean ± SD)
ADNI https://adni.loni.usc.edu	1,835	9,122	72.5 ± 5.9
CamCan https://camcan-archive.mrc-cbu.cam.ac.uk/dataaccess/	455	455	62.2 ± 13.4
CoRR http://fcon_1000.projects.nitrc.org/indi/CoRR/html/	176	176	61.6 ± 9.7
DLBS http://fcon_1000.projects.nitrc.org/indi/retro/dlbs.html	187	187	63.6 ± 13.7
IXI http://brain-development.org/ixi-dataset/	275	275	57.3 ± 10.4
OASIS3 https://www.oasis-brains.org	1,093	2,154	70.8 ± 9.0
United Kingdom Biobank https://www.ukbiobank.ac.uk	13,419	13,907	63.6 ± 7.5

Subjects were included in this study if: (1) a diagnosis on dementia status was available: healthy, mild cognitive impairment or dementia, (2) if the presence of other neuropathologies could be excluded, (3) if their age at scan time was over 40 years old. Supplementary Table 1 lists the exclusion conditions of patients form the United Kingdom Biobank (Cox et al., 2019). The data of a total of 17,440 unique subjects were collected, which represented a total of 26,276 T1-weighted MRI accounting for longitudinal acquisitions. Table 1 summarizes the number of unique subjects, scans, and their age for each dataset. A diagnosis was assigned for each MR acquisition and therefore there is as many diagnosis labels as scans. Subjects were healthy for 19,610 scans, were diagnosed as MCI for 4,505 scans and diagnosed with Alzheimer’s disease dementia (ADD) for 5,206 scans.

To ensure a good generalization of brain age predictions, all included subjects were split into three independent datasets. A training set, composed of 11,523 unique healthy subjects picked at random among the subjects who had only one acquisition, a validation set containing 2,881 unique healthy subjects who also had only one acquisition, and the testing set included all remaining subjects. The split was performed at the subject level to prevent data leakage contamination (Wen et al., 2020).

The training and validation sets were solely used for training our networks. Once networks reached an accuracy that was deemed satisfactory, brain age was estimated for each scan of the testing which appeared as new, never seen data to our networks.

The testing set was solely used to generate Structure Age statistics (see Table 2). This set included 3,036 unique subjects for a total of 11,872 scans. Structure Age was assigned to each scan independently.

**TABLE 2:** Composition of the testing set, which was independent of the training set and was used to generate all results.

Diagnosis	Number of scans	Age at scan (mean ± SD) [range]
Healthy	5,206	71.75 ± 9.0 [42.66–95.70]
MCI	4,505	74.5 ± 7.7 [50.29–97.02]
AD	2,161	76.1 ± 7.6 [50.35–95.58]

### Data preparation

The overall steps for the data preparation were detailed in our previous work (Wu et al., 2020; Besson et al., 2021). All T1-weighted MRI were processed with Freesurfer (v6.0^2^) using Northwestern University’s High Performance Computing Cluster (QUEST^3^). Preprocessing steps included bias field correction, intensity normalization, spatial normalization, skull stripping and tissue segmentation (Dale et al., 1999). The inner cortical surface, matching the white matter/gray matter junction, and the outer cortical surface, matching the gray matter/cerebro-spinal fluid interface, were then extracted. The surfaces were corrected for possible topological defects, inflated and parameterized (Fischl et al., 1999). Seven subcortical structures per hemisphere were automatically segmented using Freesurfer (amygdala, nucleus accumbens, caudate, hippocampus, pallidum, putamen, thalamus) and then modeled into surface meshes using SPHARM-PDM^4^. These structures were selected based on their size and contrast profile on T1 weighted imaging. All surfaces (inner and outer cortical surfaces and subcortical surfaces) were inflated, parameterized and registered to a corresponding surface template using a rigid-body registration to preserve the anatomy of the cortex and subcortical structures (Besson et al., 2014). This method was selected due to its demonstrated efficacy for morphological analyses (Castillo-Barnes et al., 2022).

Surface templates were converted to graphs based on their triangulation scheme (see Supplementary Figure 1). Nodes of the graphs were surface vertices, and edges of the graphs were segments across vertices. Overall, the graphs including all structures had 47,616 nodes, 32,768 from the cortical surfaces and 14,848 from the subcortical surfaces (see Table 3 for the number of nodes for each structure). Input features of the network were defined as the Cartesian coordinates of surface vertices in subjects’ native space resampled into the surface templates, centered around the origin (0, 0, 0) and divided by 100. Consequently, cortical nodes were assigned 6 features (X, Y, Z of both the inner and outer cortical surface vertices) and subcortical nodes had 3 features (X, Y, Z of subcortical surface vertices).

**TABLE 3:** Number of nodes for each structure.

Structure	Number of nodes per hemisphere
Accumbens	256
Amygdala	512
Caudate	1,024
Hippocampus	2,048
Pallidum	512
Putamen	1,024
Thalamus	2,048
Cortex	16,384

### Graph convolutional neural networks

Cortical and subcortical meshes were converted to graphs, which provide a convenient representation of their shape. This, however, comes at the expense of using special convolutional operators capable of handling data mapped on graphs instead of traditional regular grids as it is the case with 2D or 3D images. For this purpose, and similarly to our previous work (Wu et al., 2020; Besson et al., 2021), we used the graph convolutional layers introduced in Defferrard et al. (2016). In brief, this approach allows convolution filters to be learnt on unstructured data such as graphs using finite support recursive Chebychev filters applied to underlying Laplacian matrix. This approach presents the advantage to be computationally efficient and uses local information in the same way as traditional 2D or 3D CNNs.

Using these convolutional layers, the principal difference with traditional CNN architectures is the pooling operator since unstructured data such as graphs don’t possess a natural arrangement such as images. To overcome this issue, we used a multiscale binary partitioning of the cortical and subcortical meshes (Wu et al., 2020). This ensures that, for all scales *S*, graph nodes *n*_*2i–1*_ and *n*_*2i*_ with 1≤*i*≤*N*_*S*_/2 and *N*_*S*_ is the number of graph nodes at scale *S*, are neighbors and to be pooled together using a 1D pooling operator (more details in Supplementary Figure 2). The total number of graph nodes at scale 0, *N*_*0*_, is therefore a parameter of the network and was picked so that it can be successively divided by 2 and the average Euclidean distance across neighbor nodes was less than 3 mm for the cortical meshes and less than 2 mm for the subcortical meshes.

### Network architecture

**FIGURE 1:**
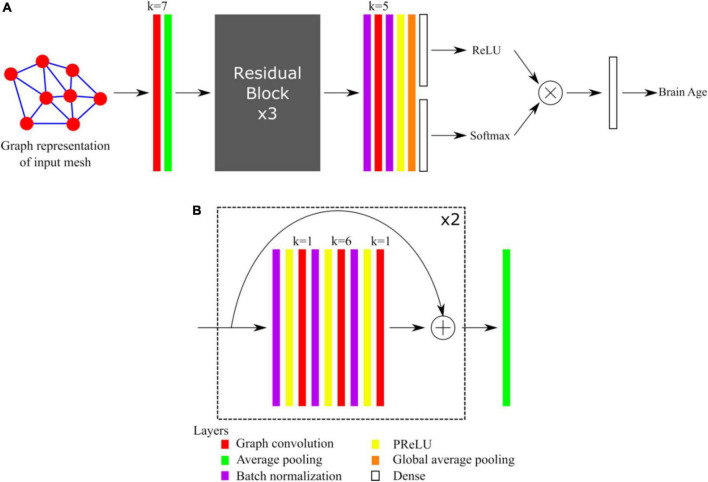
**(A)** Overall architecture of the network. **(B)** Detail of Residual Blocks. For each convolutional layer (red), the kernel size k is indicated.

The architecture of our graph convolutional neural network was based on the Residual Network architecture (He et al., 2016). Such architectures generally provide good performances, avoid the problem of vanishing gradients, and allow the training of very deep networks. We recently demonstrated that shortcut connections, as in Residual Networks architectures, improved the performances of gCNNs for the shape analysis of the subcortical structures (Azcona et al., 2021). The overall network architecture is illustrated in Figure 1A whereas the details about residual blocks are shown in Figure 1B. Our network uses a standard residual architecture with skipped connections in the residual blocks. Similarly to the Inception architecture (Szegedy et al., 2015) a first convolutional layer with a large receptive field is immediately followed by a pooling layer to reduce the dimension of the input and accelerate the training process without loss of performance. This was followed by three residual blocks, then by batch normalization, convolutional and global average pooling layers (GAP). Two independent dense layers used the output of the GAP layer: a dense layer followed by a ReLU activation aimed assigning ages to bins, whereas a dense layer followed by a Softmax activation aimed at assigning probabilities to the bins. This is in line with the recently published state-of-the-art network to estimate brain age (Peng et al., 2021), except that in our case we also made the centers of the bins learnable. Finally, the results obtained after the ReLU and Softmax activations were multiplied together and linearly combined using a last dense layer to provide the brain age estimate. The networks were implemented using Keras and Tensorflow 2.1 backend and Python 3.6.

### Network parameters and training

Ten structure-specific networks were trained: one network using all the structures together as inputs (cortical and subcortical), one network using only the cortex, one network with all the subcortical structures together only, and one network for each of the seven subcortical structures. Motivated by previous work demonstrating the value of network ensemble in brain age prediction (Levakov et al., 2020; Peng et al., 2021), twenty versions of each of the ten structure-specific networks were obtained using different seeds, therefore the total number of networks trained was 200. All networks had the same training parameters: convolutional layers had 64 filters except the very last convolutional layer which had 128 filters. Both dense layers had 75 units. The loss function was the mean squared error (MSE), the mean absolute error (MAE) was also monitored during training and validation. The optimization was done with the RMSprop algorithm with an initial learning rate set to 0.001. The learning rate was divided by 10 whenever the MAE on the validation set was not improved after 15 epochs, and the training was stopped if no improvement was obtained after 30 epochs. Networks were regularized with their L2 norm (λ_*L*2_ = 10^−12^) and a dropout of 0.75 was applied to the dense layer of the Softmax branch.

To improve the generalizability of our models, we added a data augmentation technique before inference by applying randomized rotations within ± 15 degrees during training, and ± 5 degrees during validation. Moreover, test time augmentation (TTA) was set to 3 on the validation set to improve the accuracy and robustness of the predictions (Wang G. et al., 2019). To account for prediction bias (Beheshti et al., 2019; de Lange et al., 2019; Smith et al., 2019; Peng et al., 2021), prediction biases were estimated using local weighted regressions (LOWESS) for each structure using data from the validation set. Then, LOWESS curves were parametrized using piecewise smoothing splines (smoothing parameter set to 0.1).

### Structure age prediction of the testing set

Brain age was predicted independently for each scan of the testing set, which have never been seen previously by the networks. The accuracy and robustness of the predictions were improved using TTA = 20 with the same data augmentation parameters as for the validation set, so that 400 brain age estimates were generated for each scan and each of the ten input structure combinations. All age predictions were averaged, and final age estimate was obtained after applying the piece-wise linear regression. The accuracy of structure age predictions was obtained by calculating the mean absolute error, the median absolute error, and the Person’s coefficients of correlation (r) between the predicted and actual age of the first acquisitions of healthy subjects in the testing set.

### Statistical analysis

#### Cross-sectional analysis of baseline scans

A linear fixed effect was implemented to determine the effect of cognitive status on structure age prediction. Structure-wise age prediction along with diagnosis of all baseline scans were included in this analysis to fit the following model:

$$P\,A_{S,i}=\beta_{S,0}+\beta_{S,1}\,A\,g\,e_{i}+\beta_{S,3}\,D\,i\,a\,g\,n\,o\,s\,i\,s_{i}+\varepsilon_{S,i}$$

Where *PA*_*S,i*_ is the predicted age for the structure *S*, patient *i*; *Age*_*i*_ is the real age at the baseline acquisition of patient *i*; *Diagnosis*_*i*_ is the diagnosis (healthy, MCI or ADD) of patient *i* at baseline acquisition; *β*_*S,0*_, *β*_*S,1*_ and *β*_*S,3*_ are structure-specific fixed effects coefficients and *ε*_*S,i*_ the residuals. This model seeks to answer the following question: given two subjects with the same real age, how does the diagnosis affect predicted age? All *p*-values were corrected for multiple comparisons using false discovery rate (FDR) (Benjamini and Hochberg, 1995).

#### Longitudinal analysis of structure age

A linear mixed effect model was implemented to examine the effect of cognitive status change (healthy subject, MCI or ADD) on the pace of structure aging from a morphological appearance perspective. The linear model aimed at answering the question: is there a relationship between the pace of structure aging and change in cognitive status over time, i.e., do structures age slower/faster in healthy subjects converting to MCI or ADD, as defined by their morphological appearance over time? For this purpose, we only included subjects with repeated scans, whose diagnosis at baseline scan was healthy and the age at last scan was at least 70 years old. For these subjects, all other scans along with the diagnosis were collected and subjects were grouped into one of the three categories: (1) Non-converters: baseline and last diagnosis are healthy; (2) MCI converters: baseline diagnosis is healthy and the last diagnosis is MCI; (3) ADD converters: baseline diagnosis is healthy and the last diagnosis is ADD. Using these data, the following model was fit:

$$P\,A_{S,i,j}=\beta_{S,0}+\beta_{S,1}\,A\,g\,e_{i,0}+\beta_{S,2}\,\left(D\,e\,l\,t\,a\,\_\,s\,c\,a\,n_{i,j}\times C\,o\,n\,v\,e\,r\,t\,\_\,t\,y\,p\,e_{i}\right)+b_{S,0,i}+b_{S,1,i}\,D\,e\,l\,t\,a\,\_\,s\,c\,a\,n_{i,j}+\varepsilon_{S,i,j}$$

Where *PA*_*S,i,j*_ is the predicted age for the structure *S*, patient *i* and acquisition *j*; *Age*_*i,0*_ is the real age at baseline scan for patient *i*; *Convert*_*type*_*i*_ is the type of conversion (healthy to healthy, healthy to MCI or healthy to AD) of patient *i*; *Delta*_*scan*_*i*,*j*_ is the duration between baseline scan and the *j*-th acquisition of patient *i*; *β*_*S,0*_, *β*_*S,1*_ and *β*_*S,2*_ are structure-specific fixed effects coefficients, *b*_*S,0,i*_ is a structure-specific random intercept, *b*_*S,1,i*_ a structure-specific random slope and *ε*_*S,i,j*_ the residuals. Aging slopes for MCI and AD were defined for all structures as the ratios between the fixed-effect coefficients *β*_*S,2*_ associated with MCI or AD with that associated with HC. All *p*-values were corrected for multiple comparisons using false discovery rate (FDR) (Benjamini and Hochberg, 1995).

## Results

### Accuracy of structure age predictions

**FIGURE 2:**
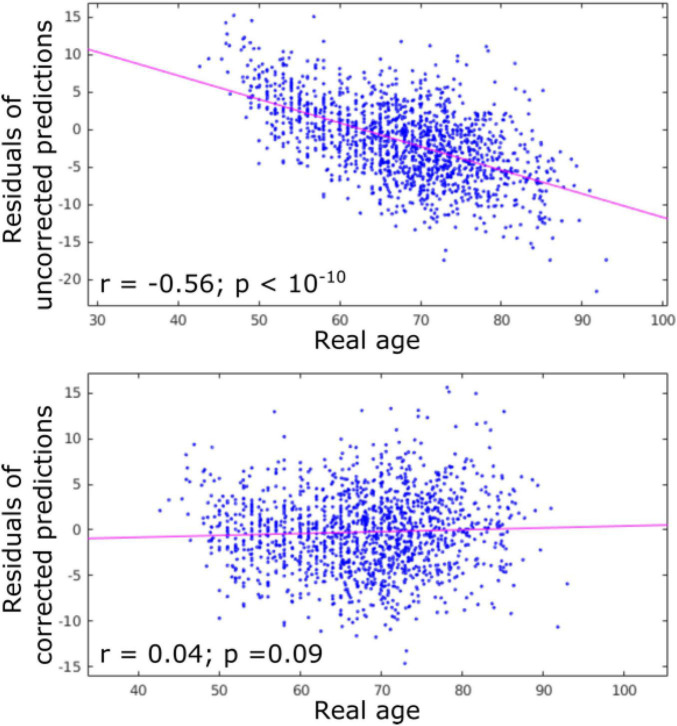
Residuals of age predictions for all structures (cortex + subcortical) before and after bias correction. Bias correction effectively suppressed the effect of real age on the predicted age.

**FIGURE 3:**
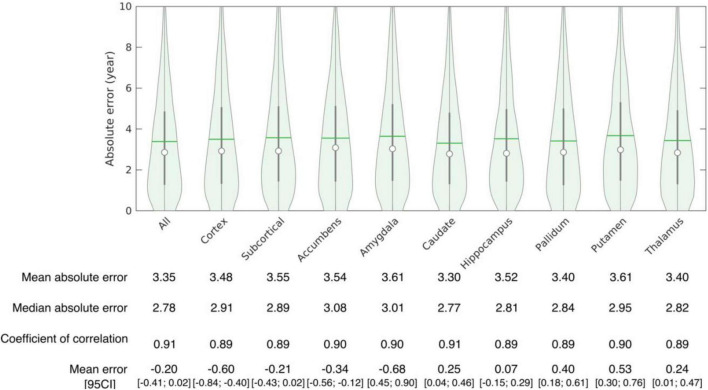
Accuracy of structure age prediction using the baseline acquisition of healthy subjects (95 CI: 95% confidence interval).

The accuracy of age prediction for each structure using the baseline scans of healthy controls from the independent testing set as well as the bias correction effectiveness are summarized in Figures 2, 3. Age was well estimated for all structures, the mean absolute error ranging from 3.30 years when using the caudate only, to 3.61 years when using the amygdala only or the putamen only. For all structures, the coefficient of correlation between the actual and the predicted structure age was at least 0.89.

### Cross-sectional analysis of baseline scans

**FIGURE 4:**
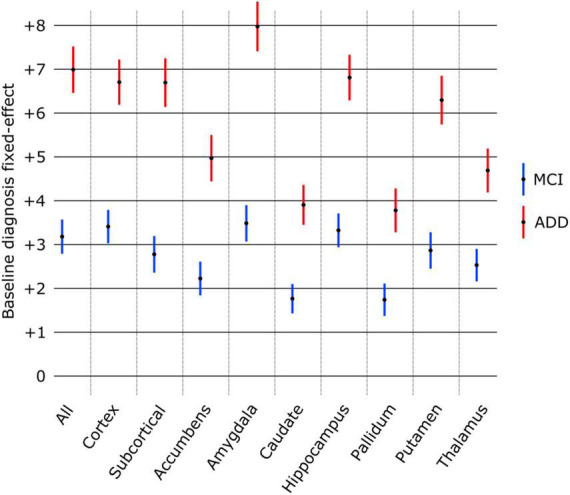
Effect of baseline diagnosis on structure age prediction (point estimate and 95% confidence intervals). The predicted age was significantly larger (*p* < 0.0001 FDR corrected) for all structures compared to healthy controls (group of reference, no effect) as well as between MCI and ADD groups.

The effect of diagnosis at baseline on structure age prediction is shown in Figure 4. Using only the baseline scans and diagnosis of all subjects, the predicted age for all structures was found significantly larger in patients with MCI and ADD compared to healthy subjects. The amygdala was found to have the largest effect as MCI patients were estimated to be 3.48 years older, and ADD patients 7.97 years older than healthy controls. On the other hand, the pallidum had the smallest effect as pallidum age was estimated to be 1.74 years older in MCI patients and 3.78 years older in ADD patients compared to healthy controls.

### Longitudinal analysis of structure age

The results of the mixed effect linear model for the longitudinal analysis of structure age predictions are presented in Table 4. Compared to healthy controls, MCI converters have significantly increased estimated age for the composite subcortical structures, as well as individual amygdala, caudate, hippocampus, pallidum and putamen. ADD converters have significantly increased estimated age for the whole brain (cortical + subcortical), composite subcortical structures, as well as individual accumbens, amygdala, hippocampus, pallidum and putamen. Overall, the aging pattern in ADD converters is similar but consistently more marked than the aging pattern of MCI converters compared to healthy controls, except for the caudate, found to be significantly increased in MCI converters only.

**TABLE 4:** Effects of cognitive status conversion on structure age.

Model Term	Cortical + subcortical	Cortex	Subcortical	Accumbens	Amygdala	Caudate	Hippocampus	Pallidum	Putamen	Thalamus
MCI converter fixed effect	+ 0.93	+0.78	** + 1.20**	+0.33	** + 1.34**	**+1.00**	** + 1.83**	**+1.13**	** + 1.39**	+0.78
ADD converter fixed effect	** + 2.29**	+1.52	** + 2.56**	**+2.08**	** + 2.86**	+1.32	** + 3.18**	**+2.08**	** + 3.07**	+1.59
MCI converter delta scan interaction fixed effect	** + 12.28%**	+3.45%	+ 6.53%	+12.22%	+ 7.99%	+2.52%	+ 7.74%	−0.31%	−0.75%	+ 1.40%
ADD converter delta scan interaction fixed effect	** + 31.16%**	**+38.08%**	+ 20.46%	**+25.57%**	** + 35.84%**	+6.18%	** + 18.59%**	**+19.11%**	+ 8.87%	−3.20%

Significant differences with non-converters are displayed in bold font (*p* < 0.05, FDR corrected).

The aging pace, determined by the fixed effect term between the type of conversion and the time across scans (delta scan), was found largely and widespread increased in ADD converters compared to healthy controls with significantly faster aging affecting the whole brain, cortex, accumbens, amygdala, hippocampus and pallidum. A similar pattern of widespread increased aging pace was identified in MCI converters, although to a lesser degree and included only significant whole brain findings.

## Discussion

### Successful prediction of brain age using structure-wise analysis

Using baseline MRI scans from healthy controls, accurate brain age was predicted using a combination of all brain structures combined as well as each individual brain structure in isolation. The accuracy of our results are equivalent or superior to those of others when taking the whole brain as a predictive model (Cole et al., 2017; Liem et al., 2017; Beheshti et al., 2022). The strong performance of each subcortical structure in isolation is notable. While this is a novel finding, it is not altogether unexpected due to the adjacency of the subcortical structures to the ventricles. Given the previously well-described relationship between the ventricles and brain age, this finding is congruent with previous reports (Scahill et al., 2003; Preul et al., 2006). Ours is the first work to investigate structure-wise prediction of brain age using a gCNN approach. While findings were relatively consistent across individual structures and when taking all structures together, variability across structures was identified. These findings suggest a unique but tandem contribution to brain aging exerted by each individual brain structure. Our findings utilizing a gCNN approach, corroborate those of others using non-graph based methods which have demonstrated enhanced brain age prediction when accounting for a combination of both local and regional metrics in the prediction algorithm (Beheshti et al., 2022).

### Differences identified between healthy and pathologic aging populations

When comparing findings on baseline MRI between healthy and pathologic aging populations, including those with MCI and ADD, structure-wise analysis continues to reveal novel insights into these different populations. All brain structures analyzed together and individually revealed a consistent relationship between healthy, MCI and ADD populations including a modest increased deviation of predicted brain age in MCI and a more marked increased deviation of predicted brain age in ADD, both of which were significant compared to healthy controls and between pathologic entities.

The most notable increased deviations from chronologic age were found in the amygdala with an overestimation of brain age by nearly 3.5 and 8 years in MCI and ADD populations respectively, when predicting brain age from the surface of the amygdala alone. This was followed by an overestimation of brain age by analysis of the cortical ribbon alone by 3.4 and 6.7 years for MCI and ADD populations respectively, while the analysis of the hippocampus alone yielded an overestimation of brain age by 3.3 and 6.8 years for MCI and ADD populations, respectively.

The significant overestimation of structure-wise brain age in pathologic aging populations suggests a prominent role may be played by each of these individual brain structures in driving altered aging patterns. The findings involving the cortex and hippocampus are unsurprising in our current understanding of aging, however, the substantial role of the amygdala and putamen in pathologic aging has been less extensively investigated and discussed.

Our findings advance the work of Cuenod, Maunoury and others following their descriptions of amygdala atrophy serving as a marker of early ADD (Cuenod et al., 1993; Maunoury et al., 1996). More recently, disordered olfaction and specifically impairment of the olfactory amygdala in ADD has been discussed and postulated to be an early driver of ADD symptom onset (Ubeda-Banon et al., 2020).

### Differential structuro-temporal evolution between groups

When comparing findings over time for individuals who remain healthy against those who convert from healthy to either MCI or ADD, gCNN analysis reveals insights into the temporal evolution of brain aging both at the whole brain and single-structure level. Specifically, individuals who convert to either MCI or ADD demonstrate an overestimated structure age (conversion fixed effect) compared to non-converters who remain healthy, but this overestimation is more modest than when statically comparing groups at a single point in time.

While the net brain age overestimations are relatively modest on these longitudinal comparisons, structure-wise gCNN analysis also provides novel temporal information regarding the pace of brain aging between groups over time. Overall, brain structures demonstrate a significantly faster pace (conversion delta scan interaction fixed effect) of brain aging within individuals converting to either MCI or ADD compared to non-converting healthy individuals with the exception of the caudate, putamen and thalamus which remain relatively aligned with healthy agers regardless of conversion status. Of the brain structures demonstrating a faster pace of brain aging in pathologic aging populations, the relative increase in aging pace was more pronounced in those converting to ADD compared to those converting to MCI. Ours is the first to represent these dynamic relationships between MCI and ADD converters at the whole brain and single structure levels.

An additional novel finding from this analysis yields overarching consistency across static and dynamic analyses. Specifically, the roles played by the cortex, hippocampus and amygdala within the pathologic aging processes characterized as MCI and ADD. Interestingly, the amygdala reveals a more pronounced static difference in brain age overestimation whereas the cortex-only model and amygdala-only model each demonstrate the most elevated pace of aging in ADD converters, specifically 38.08 and 35.84% faster rates of brain aging respectively, compared to healthy non-converters. This supports long standing work implicating the amygdala in the neurobiology of dementia given its role in both the cholinergic and serotonergic systems, long-thought to underpin the development of ADD and other dementia types (Rodriguez et al., 2012; Mesulam, 2013). This finding is also in keeping with prior work demonstrating accelerated atrophy of the amygdala in healthy adults at increased genetic risk of developing ADD (An et al., 2021) as well as in individuals with MCI and ADD (Feng et al., 2021). Taken together, these findings add to the growing body of literature that identifies a prominent role played by the amygdala in the pathophysiology of deteriorating cognitive function. Of note, the hippocampus demonstrates a relatively modest increased pace of aging in pathologic agers compared to healthy agers at 7.74% increase in MCI converters and 18.59% increase in ADD converters.

The results of this work must be interpreted within the constraints of large, publicly available database-based analyses investigating aging and cognition including the subjective criteria used to define MCI and ADD classification across multiple participating sites as well as multiple scanner and acquisition protocols used in MR data collection. Despite these limitations, we believe our findings advance our understanding of brain aging by providing a novel structuro-temporal understanding of aging in healthy and pathologic aging populations.

## Data availability statement

All data used in this analysis are available as identified in Table 1.

## Author contributions

PB and SB contributed to conception and design of the study and wrote the first draft of the manuscript. AM and ER provided subject matter input and guidance and provided critical review of the manuscript throughout its preparation. PB organized the database, performed all computational analysis, and created all figures and tables. PB and NG performed statistical analysis. HZ provided statistical input and guidance. All authors contributed to manuscript revision, read, and approved the submitted version.

## Footnotes

1
https://adni.loni.usc.edu

2
https://surfer.nmr.mgh.harvard.edu

3
https://www.it.northwestern.edu/research/user-services/quest/

4
https://www.nitrc.org/projects/spharm-pdm/
