# Supplementary material for: Geometric deep learning reveals a structuro-temporal understanding of healthy and pathologic brain aging
Source: Front Aging Neurosci. 2022 Aug 23;14:895535. doi: 10.3389/fnagi.2022.895535 (PMC9445244; doi:10.3389/fnagi.2022.895535)
Supplement: Supplementary file 1 [file Data_Sheet_1.docx]

Table S1 List of excluding conditions for subjects collected from the UK Biobank dataset. From Cox, S. R., Ritchie, S. J., Fawns-Ritchie, C., Tucker-Drob, E. M. and Deary, I. J. Structural brain imaging correlates of general intelligence in UK Biobank. Intelligence 2019; 76:101376

| **Condition** | | **Code** |
| --- | --- | --- |
|  | Dementia or Alzheimer’s disease | 1263 |
|  | Parkinson’s disease | 1262 |
|  | Chronic degenerative neurological | 1258 |
|  | Guillain-Barré syndrome | 1256 |
|  | Multiple sclerosis | 1261 |
|  | Other demyelinating disease | 1397 |
|  | Stroke or ischaemic stroke | 1081 |
|  | Brain cancer | 1032 |
|  | Brain haemorrhage | 1491 |
|  | Brain / intracranial abscess | 1245 |
|  | Cerebral aneurysm | 1425 |
|  | Cerebral palsy | 1433 |
|  | Encephalitis | 1246 |
|  | Epilepsy | 1264 |
|  | Head injury | 1266 |
|  | Infections of the nervous system | 1244 |
|  | Ischaemic stroke | 1583 |
|  | Meningeal cancer | 1031 |
|  | Meningioma (benign) | 1659 |
|  | Meningitis | 1247 |
|  | Motor neuron disease | 1259 |
|  | Neurological injury / trauma | 1240 |
|  | Spina bifida | 1524 |
|  | Subdural haematoma | 1083 |
|  | Subarachnoid haemorrhage | 1086 |
|  | Transient ischaemic attack | 1082 |


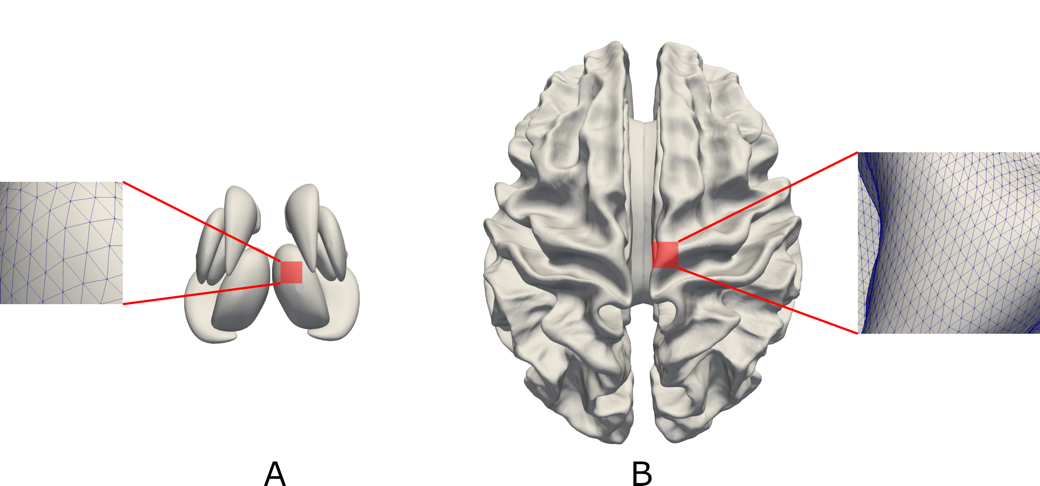


**Figure S1** Visualization of the surface template for the subcortical structures (A) and the cortex(B), with close up on their high-density triangulations. Template surface meshes were converted to graphs using their triangulation schemes. Nodes of the graphs were defined as the surface vertices, and edges of the graphs were triangles segments across vertices. A weight $W_{i,j}$ was assigned to the edge between neighbor nodes $i$ and $j$ such as:

$$W_{i,j}=\frac{e^{\frac{-{d_{i,j}}^{2}}{2}}}{\sqrt{2\pi}}$$

with $d_{i,j}$ is the Euclidean distance between the nodes $i$ and $j$.


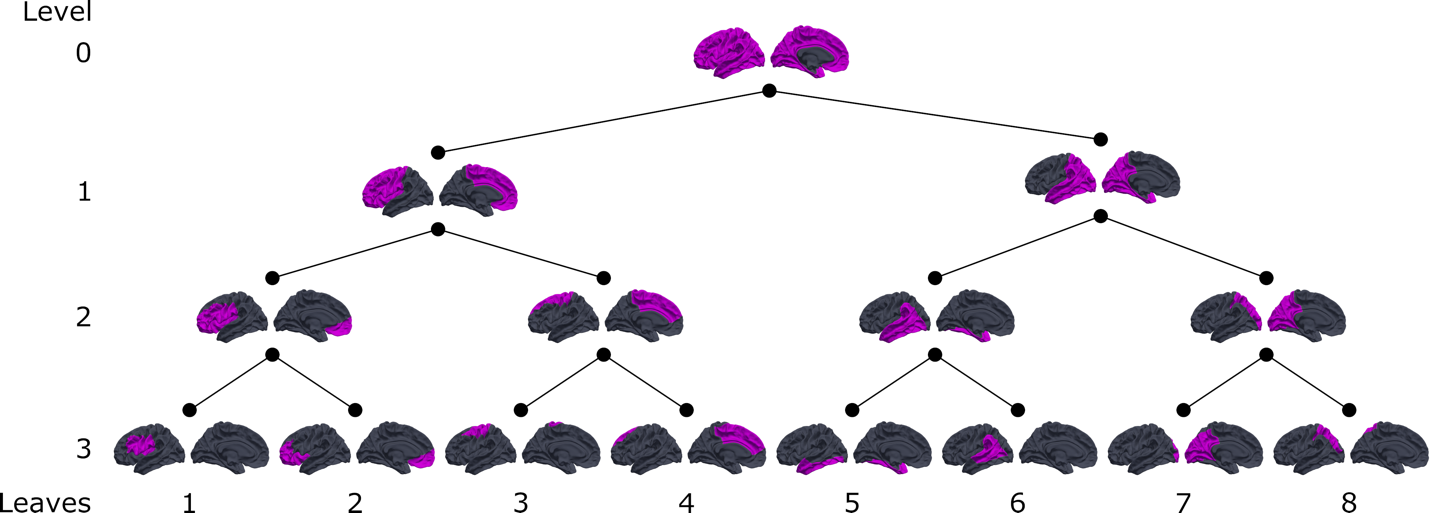


Figure S2 First four level of the hierarchical decomposition of the left cortical surface. The initial level (0) is the whole structure, in that case the left cortical surface masking out non-cortical regions such as Freesurfer’s medial wall and parahypocampal regions.

High-resolution template graphs underwent a hierarchical dichotomic partitioning using the following steps:

1. For each structure, define the root leaf as the graph corresponding to the high-density mesh
2. Repeat these steps until the average distance across neighbor leaves is less than a threshold $T$
   1. Given a set of leaves at level $L$, partition each leaf at level $L$ into two child leaves at level $L+1$ using spectral clustering (Von Luxburg, 2007). The new leaves are therefore subgraphs of their parent leaf;
   2. Order the $2^{L+1}$ leaves of level $L+1$ such that the leaves $2\left( i-1 \right)$ and $2i$ are partitions of leaf $i$ at level $L$;
   3. For each leaf of level $L+1$, identify its center node as the node whose betweenness centrality is largest (Rubinov & Sporns, 2010);
   4. Defines the partition neighbor matrix $M_{L+1}$, of size $2^{L+1}\times2^{L+1}$, such that

$$M_{L+1}\left( i,j \right)=\left\{ \begin{matrix} W_{L+1}\left( i,j \right) & \text{if leaves }\text{i }\text{ and }\text{j }\text{ have neighbor vertices on the mesh} \\ 0 & \text{if }\text{i}\text{ =}\text{j} \\ 0 & \text{otherwise} \end{matrix} \right.$$

and

$$W_{L+1}\left( i,j \right)=\frac{e^{\frac{-{d_{i,j}}^{2}}{2}}}{\sqrt{2\pi}}$$

where $d_{i,j}$ is the geodesic distance (along the mesh) between the center of the leaf *i* and the center of the leaf *j* at level $L+1$;
   5. Compute the average distance across leaves’ centers and exit the loop if less than $T$, otherwise continue to step a.

The average distance threshold $T$ was set to 3 mm for the cortical surfaces and 2 mm for the subcortical surfaces. Therefore, the number of decomposition levels for each structure was a function of their surface area.
